# Supplementary material for: Ultrasensitive Detection of Interleukin 6 by Using Silicon Nanowire Field-Effect Transistors
Source: Sensors (Basel). 2023 Jan 5;23(2):625. doi: 10.3390/s23020625 (PMC9865274; doi:10.3390/s23020625)
Supplement: Supplementary file 1 [file sensors-23-00625-s001.zip › sensors-2067084-supplementary.pdf]

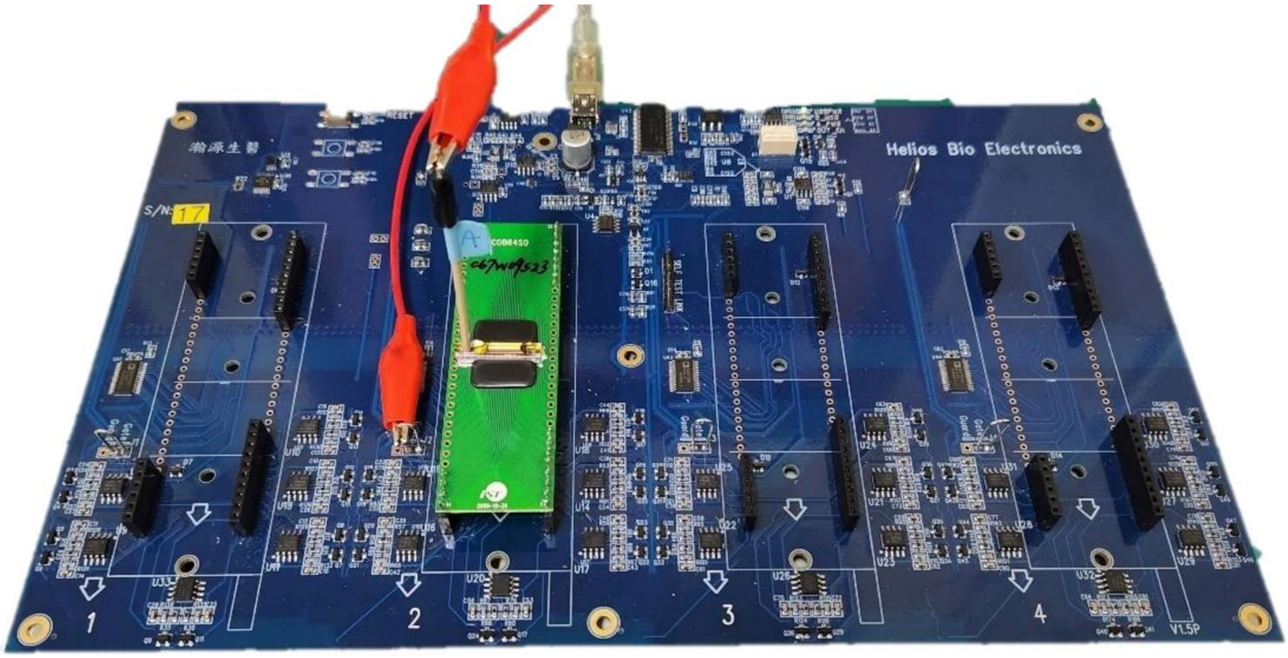

(a)

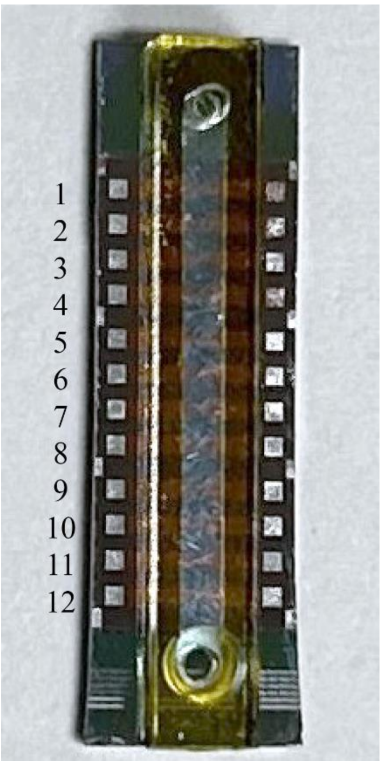

(b)

Figure S1. The packaged chip-on-board (COB) SiNW-FET is installed into an electrical circuit board for signal recording. (a) The overview of the circuit board and the packaged COB SiNW-FET. The V_g is applied using an electrode which is connected with the electrical board via a red wire. (b) Detailed features of the packaged COB SiNW-FET. It contains 12 SiNW-FET devices (24 nanowires), and the signals from 12 channels are recorded in the experiments (Ch1 = device 1, Ch2 = device 2, and so forth. The signal is generated from 2 nanowire channels for each SiNW-FET device.).

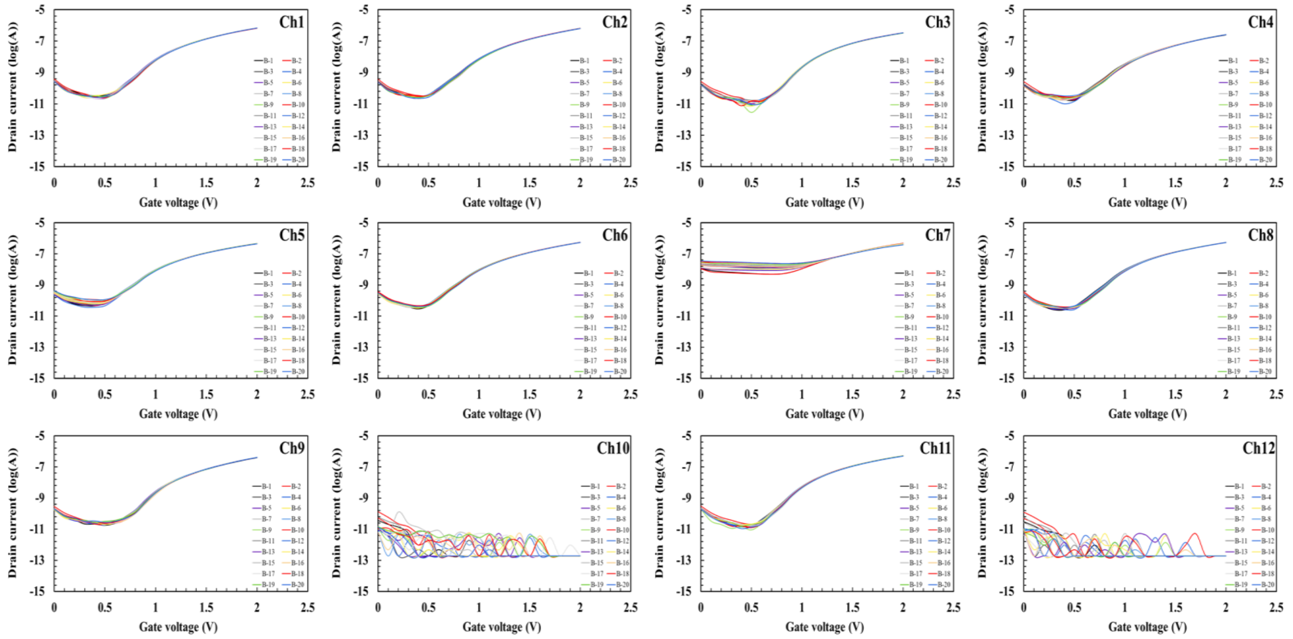

Figure S2. The stability test of the SiNW-FET devices without any modification. The test is conducted in the 50 mM BTP buffer (pH 7.4), and the measurement is repeated twenty times. Some devices have lousy quality, like channels 7, 10, and 12. By calculating the data obtained from other channels, the average values of the shift in gate voltage and standard deviation at the drain current (I_d) of 1×10^{-9} A are 0.2 mV and 5.5 mV, respectively.

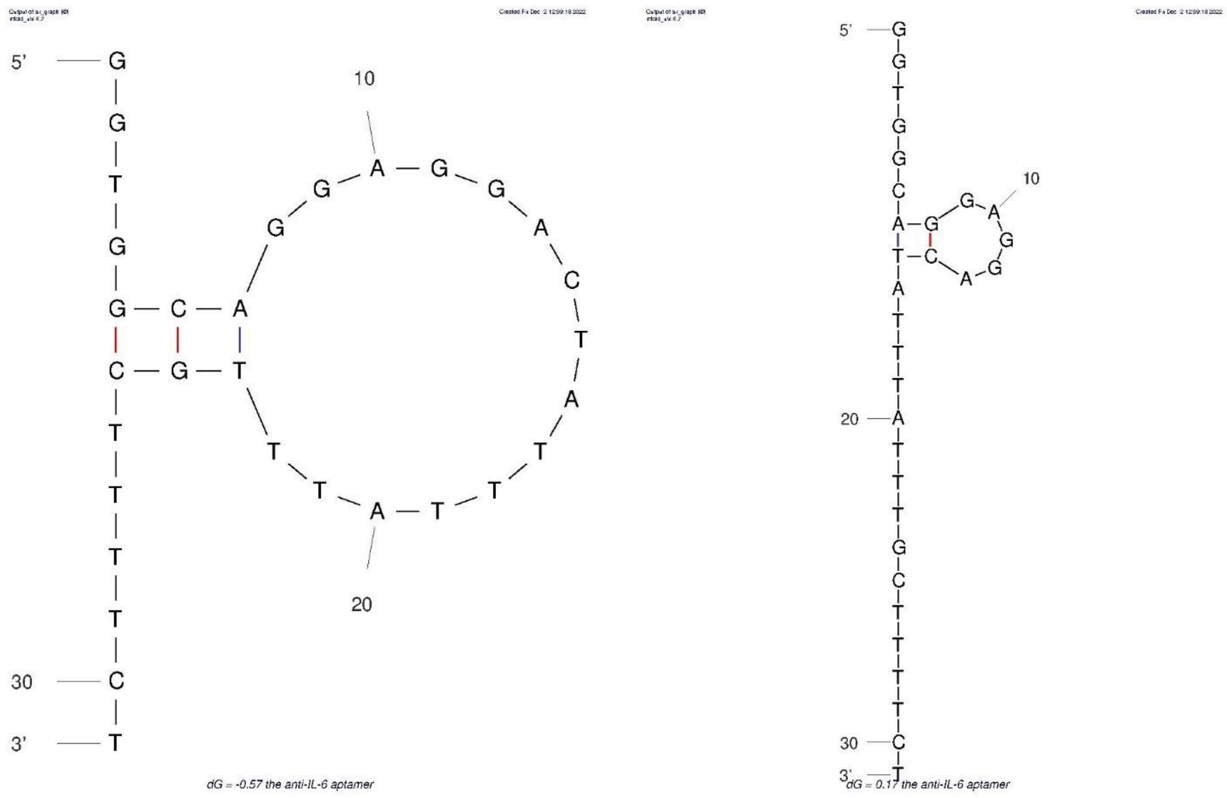

(a) (b)

Figure S3. The two structures of the anti-IL-6 aptamer are predicted by using the Mfold web server. The lengths of two structures in the vertical direction are calculated based on the number of bases are (a) 4.08 nm, and (b) 8.16 nm.

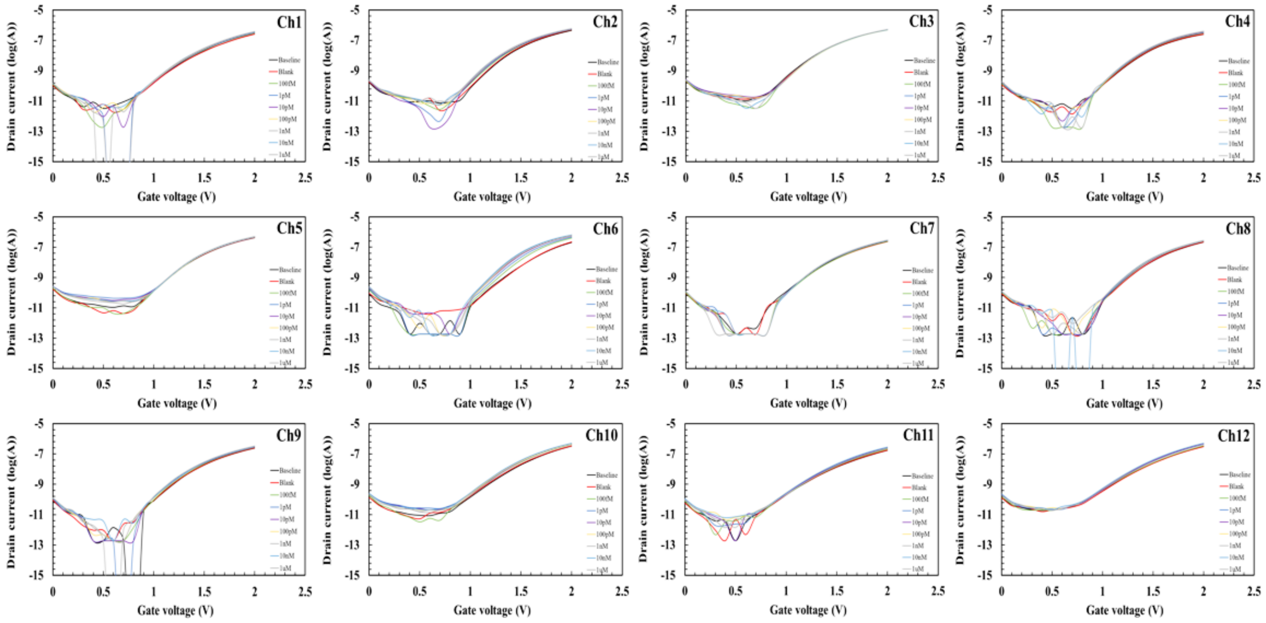

Figure S4. The $I_d - V_g$ curves obtained from twelve channels of the SiNW-FET by using the aptasensor. The quality of each nanowire is not identical, and some of the nanowires have lousy quality, like channels 1, 8 and 9.

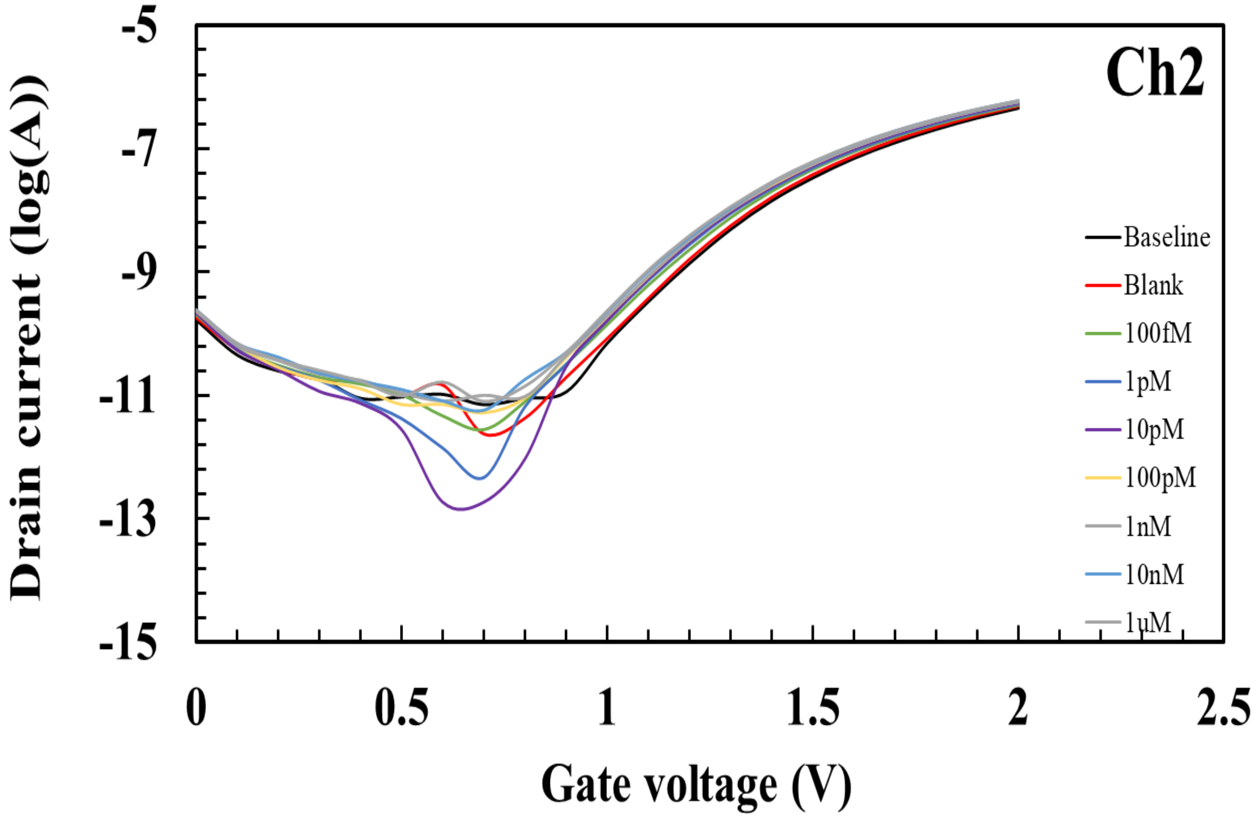

Figure S5. The enlargement image of recorded curves in channel 2 of Figure S4.

Table S1. Five examples of raw data for the detection of IL-6 on the SiNW-FETs with APTES-GA-Ab layer.

Groups	ch1 (mV)	ch2 (mV)	ch3 (mV)	ch4 (mV)	ch5 (mV)
Blank	-26.1241	-26.8943	-10.9187	-15.7991	-12.75717
21 pg/mL (1 pM)	-39.1889	-44.3952	-18.7305	-26.1448	-15.3422
210 ng/mL (10 pM)	-50.0620	-49.4864	-21.687	-37.2942	-18.5127
2.1 ng/mL (100 pM)	-48.3429	-49.8205	-19.9994	-34.8226	-19.6162
21 ng/mL (1 nM)	-50.9766	-51.8415	-25.7842	-35.8480	-22.6979
210 ng/mL (10 nM)	-51.5569	-53.0490	-35.9350	-48.3660	-27.5862

Table S2. Five examples of raw data for the detection of IL-6 on the SiNW-FETs with APS-GA-Ab layer.

Groups	ch1 (mV)	ch2 (mV)	ch3 (mV)	ch4 (mV)	ch5 (mV)
Blank	-14.9595	-10.5056	-6.8901	-2.94781	-15.7302
21 pg/mL (1 pM)	-18.3867	-18.4882	-10.3212	-10.3178	-17.6109
210 ng/mL (10 pM)	-21.2999	-19.5811	-17.5142	-14.9990	-20.6079
2.1 ng/mL (100 pM)	-23.2895	-24.4332	-21.0025	-19.5168	-24.8676
21 ng/mL (1 nM)	-25.8483	-26.1696	-21.5488	-18.8569	-29.2589
210 ng/mL (10 nM)	-24.9494	-23.2263	-27.5813	-25.6442	-29.9680

Table S3. Five examples of raw data for the detection of IL-6 on the SiNW-FETs with mSAMs-GA-Ab layer.

Groups	ch1 (mV)	ch2 (mV)	ch3 (mV)	ch4 (mV)	ch5 (mV)
Blank	-9.8697	-15.3840	-24.6308	-1.9136	-1.4856
21 pg/mL (1 pM)	-19.5492	-22.1176	-40.2115	-10.5291	-6.2969
210 ng/mL (10 pM)	-36.3453	-26.2015	-62.5557	-39.6697	-16.9319
2.1 ng/mL (100 pM)	-46.7271	-32.5584	-75.8925	-76.4369	-39.0368
21 ng/mL (1 nM)	-61.3980	-27.8809	-84.6234	-92.1436	-58.7537
210 ng/mL (10 nM)	-63.1347	-39.4248	-81.3974	-95.4557	-51.9578

Table S4. Results of one-way analysis of variance (ANOVA) with Tukey's HSD Test for detecting various concentrations of Interleukin-6 on the SiNW-FETs with the mSAMs-GA-Apt layer.

(I)Groups	(J)Groups	Mean Difference (I-J)	Std. Error.	Sig.
2.1 pg/mL **(100 fM)**	21 pg/mL**(1 pM)**	13.47412*	4.47405	.039
	210 pg/mL**(10 pM)**	19.56948*	4.47405	.000
	2.1 ng/mL **(100 pM)**	23.66395*	4.47405	.000
	21 ng/mL **(1 nM)**	38.86775*	4.47405	.000
	210 ng/mL **(10 nM)**	39.57250*	4.47405	.000
21 pg/mL**(1 pM)**	2.1 pg/mL **(100 fM)**	-13.47412*	4.47405	.039
	210 pg/mL**(10 pM)**	6.09536	4.47405	.749
	2.1 ng/mL **(100 pM)**	10.18983	4.47405	.215
	21 ng/mL **(1 nM)**	25.39363*	4.47405	.000
	210 ng/mL **(10 nM)**	26.09838*	4.47405	.000
210 pg/mL**(10 pM)**	2.1 pg/mL **(100 fM)**	-19.56948*	4.47405	.001
	21 pg/mL**(1 pM)**	-6.09536	4.47405	.749
	2.1 ng/mL **(100 pM)**	4.09447	4.47405	.942
	21 ng/mL **(1 nM)**	19.29827*	4.47405	.001
	210 ng/mL **(10 nM)**	20.00302*	4.47405	.000
2.1 ng/mL **(100 pM)**	2.1 pg/mL **(100 fM)**	-23.66395*	4.47405	.000
	21 pg/mL**(1 pM)**	-10.18983	4.47405	.215
	210 pg/mL**(10 pM)**	-4.09447	4.47405	.942
	21 ng/mL **(1 nM)**	15.20380*	4.47405	.013
	210 ng/mL **(10 nM)**	15.90855*	4.47405	.008
21 ng/mL **(1 nM)**	2.1 pg/mL **(100 fM)**	-38.86775*	4.47405	.000
	21 pg/mL**(1 pM)**	-25.39363*	4.47405	.000
	210 pg/mL**(10 pM)**	-19.29827*	4.47405	.001
	2.1 ng/mL **(100 pM)**	-15.20380*	4.47405	.013
	210 ng/mL **(10 nM)**	.70475	4.47405	1.000
210 ng/mL **(10 nM)**	2.1 pg/mL **(100 fM)**	-39.57250*	4.47405	.000
	21 pg/mL**(1 pM)**	-26.09838*	4.47405	.000
	210 pg/mL**(10 pM)**	-20.00302*	4.47405	.000
	2.1 ng/mL **(100 pM)**	-15.90855*	4.47405	.008
	21 ng/mL **(1 nM)**	-.70475	4.47405	1.000

*, The mean difference is significant at the .05 level.
